# Early COVID‐19 and Severity of Subsequent Omicron Infection in Ontario Canada

**DOI:** 10.1111/irv.70197

**Published:** 2026-01-12

**Authors:** Caroline Kassee, Altynay Shigayeva, Christopher Kandel, Lubna Farooqi, Zoe Zhong, Anne‐Claude Gingras, Brenda L. Coleman, Lois Gilbert, Wayne L. Gold, Maria Major, Tony Mazzulli, Samira Mubareka, Srinivas Rao Valluri, Catherine Martin, Moe H. Kyaw, John M. McLaughlin, Allison McGeer

**Affiliations:** ^1^ Department of Microbiology Sinai Health Toronto Canada; ^2^ Michael Garron Hospital, Toronto East Health Network Toronto Canada; ^3^ Department of Medicine University of Toronto Toronto Canada; ^4^ Lunenfeld‐Tanenbaum Research Institute Sinai Health Toronto Canada; ^5^ Department of Molecular Genetics University of Toronto Toronto Canada; ^6^ Dalla Lana School of Public Health University of Toronto Toronto Canada; ^7^ University Health Network Toronto Canada; ^8^ Pfizer Canada Kirkland Canada; ^9^ Department of Laboratory Medicine and Pathobiology University of Toronto Toronto Canada; ^10^ Sunnybrook Health Sciences Centre Toronto Canada; ^11^ Pfizer Inc. New York New York USA

**Keywords:** activities of daily living (ADL), Omicron, pre‐existing immunity, prior infection, reinfection, severity

## Abstract

We evaluated whether having early COVID‐19 reduced the severity of subsequent Omicron infection, assessing activities of daily living (ADL), healthcare utilization and illness duration. Comparisons were made between persons with (1) early COVID‐19‐compatible illness with a negative test, (2) early lab‐confirmed SARS‐CoV‐2 14–26 months and (3) early lab‐confirmed SAR‐CoV‐2 > 26 months before Omicron infection. Among 261 patients with laboratory‐confirmed Omicron, 177 (68%) had COVID‐19 in 2020, a median of 793 days (IQR, 659–902) prior to Omicron infection. Compared to no early COVID‐19, COVID‐19 14–26 months, but not > 26 months, before was associated with reduced impact on ADL during first Omicron infection (OR 0.52, 95% CI 0.29–0.93).

## 1 Introduction

Exposure to SARS‐CoV‐2 infections and vaccinations, combined with virological changes, has led to milder disease [1, 2, 3]. Studies have demonstrated that prior SARS‐CoV‐2 infection, in combination with vaccination, reduces the risk of reinfection with SARS‐CoV‐2 and the risk of infection‐associated hospitalization [4, 5, 6, 7]. There are fewer data, however, describing the protection that prior infection provides against other COVID‐19 outcomes like activities of daily living (ADL), healthcare resource utilization or symptom duration. There has also been limited study of the impact of previous infection on COVID‐19 severity. We aimed to assess the impact of early SARS‐CoV‐2 infection on the severity of Omicron infection.

## 2 Methods

### 2.1 Design, Setting and Population

We performed a secondary analysis of data from a prospective cohort study conducted in Toronto, Canada, to assess the impact of early COVID‐19 on subsequent Omicron infections in a highly vaccinated population [8]. For the prospective cohort study, we approached all persons hospitalized with PCR‐confirmed COVID‐19 between 15 January and 30 September 2020 in seven Toronto hospitals and randomly selected persons with PCR‐confirmed COVID‐19 diagnosed in emergency departments or COVID‐19 assessment centres between 15 January and 15 June 2020 and managed as outpatients. We also approached (a) hospitalized test‐negative controls with a COVID‐19‐compatible illness but a negative PCR test for SARS‐CoV‐2, matched individually (1:4) to COVID‐19 infected hospitalized participants on date of hospitalization and age group (+/−10 years), and (b) outpatient test‐negative controls, group‐matched (1:4) to outpatient cases of COVID‐19 by date of infection, age group and indication for testing (only travellers, close contacts of infected patients and healthcare workers were eligible for testing during this time period). Most participants had been in studies of early COVID‐19 and were enrolled in this study between September 2021 and June 2022. They were administered baseline questionnaires at enrolment and followed every 2 weeks until 31 January 2023 to identify symptomatic SARS‐CoV‐2 infections, chronic medical conditions and COVID‐19 vaccinations [8] (see Supporting Information and Supplementary Figures S1–S3 for context and details).

**FIGURE 1:**
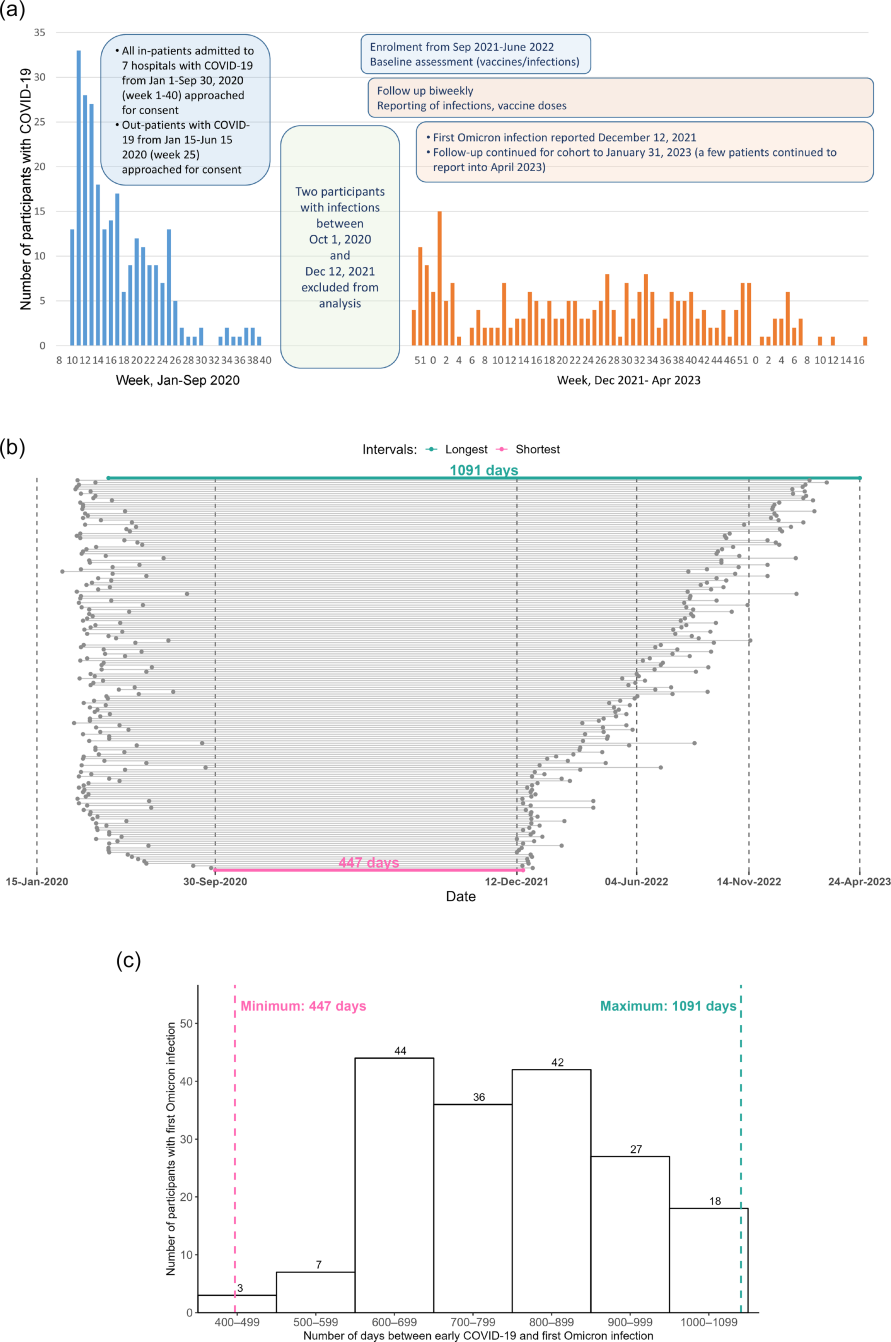
Timing of COVID‐19 infections in the study cohort. (A) Dates of early infection in cohort participants, and dates of symptomatic Omicron infections in participants. Note that two patients were excluded from analysis because of infections between 1 October 2020 and 11 December 2021. (B) Days from early infection to first Omicron infection in the 177 participants with an early infection, ordered from shortest interval at the bottom (447 days) to longest on the top (1091 days). (C) Distribution of time from early infection to first Omicron infection among study participants.

Participants were included in this analysis if their baseline and follow‐up questionnaires were complete, and they had laboratory‐confirmed SARS‐CoV‐2 infection due to Omicron between 12 December 2021 and 23 April 2023. Participants with a laboratory‐confirmed SARS‐CoV‐2 infection between 30 September 2020 and 11 December 2021 were excluded, as were participants who reported asymptomatic COVID‐19 (i.e., a positive PCR or rapid‐antigen test (RAT) result in the absence of symptoms) during the Omicron period (Figure 1).

### 2.2 Outcomes

The outcome of interest was the severity of laboratory‐confirmed symptomatic Omicron infection. Study participants completed a summary illness report that recorded the presence of fever, the impact on their ability to perform ADL, the requirement for healthcare and the duration of their illness (see Supporting Information). Three measures of severity were derived: a combination of the impact of illness on ADL and the presence/absence of fever (see Table 1 and Supporting Information); healthcare visits (none vs. ≥ 1); and the duration of illness, categorized as 0–4, 5–9, 10–14, 15–19 or ≥ 20 days (see Figure S4 for distribution of duration of illness by day).

### 2.3 Exposure

We compared the severity of symptomatic Omicron infections between three groups: those with no early infection (hospitalized or outpatients with compatible symptoms but negative test for SARS‐CoV‐2); those with early COVID‐19 infection (either hospitalized or outpatient) 14–26 months before their first Omicron infection; and those with early COVID‐19 infection (hospitalized or outpatient) more than 26 months before their first Omicron infection. Twenty‐six months (793 days) corresponded to the median time between the early COVID‐19 infection and first Omicron infection in the cohort (Figure 1).

### 2.4 Statistical Analysis

Univariate differences between the three exposure groups were compared using Chi‐squared or Fisher's Exact tests. Proportional odds ordinal regression models were fit for the fever/ADL and duration of illness outcomes. A binary logistic regression model was fit for the healthcare outcome. Adjusted models included the following covariates: age (continuous), sex (male or female), neighbourhood income quintile (by postal code mapped to census tract) [9], underlying chronic medical conditions, immunocompromising conditions [8], time from most recent vaccine dose and hand‐to‐face habits [8]. Tests for model fit and assumption violations were performed for all models. Sensitivity analyses were performed to explore the effect of early COVID‐19 on outcomes by Omicron SARS‐CoV‐2 test type (PCR or RAT), Omicron variant period (BA.1/2 [from 12 December 2021 through 3 June 2022]), BA.4/5 [from 4 June 2022 through 13 November 2022] or BA.5/BQ1.1/XBB (from 14 November 2022 through 24 April 2023) [10] and number of COVID‐19 vaccine doses. Hospital admission for early COVID‐19 was explored as an effect modifier. A secondary analysis was conducted to evaluate the association between time from early infection to Omicron reinfection and severity of Omicron infection, with time as a continuous variable. All analyses were conducted using R 4.2.3 and SAS 9.4.

## 3 Results

Of the 261 study participants who had a symptomatic Omicron infection from 12 December 2021 through 24 Apr 2023, 151 (58%) were female, and 81 (31%) had at least one underlying chronic medical condition (Table 1 and Figure 1A). Eighty‐four (32%) of these participants had not had an early SARS‐CoV‐2 infection. There was a median of 793 [IQR, 659–902] days between an early infection (i.e., before September 2020) and the first Omicron reinfection, such that 88 (34%) and 89 (34%) had an early symptomatic infection 14–26 months and > 26 months prior to their first Omicron infection, respectively (Figure 1B,C). The median age of participants was 52 (36–66), 48 (34–58) and 52 (42–63) years in the three groups. Of the 261 Omicron infections, 118 (46%), 102 (39%) and 41 (16%) occurred during the BA.1/2, BA.4/5 and BA.5/BQ1.1/XBB periods, respectively. Twelve (5%) participants had a second Omicron infection (Table S1). Among participants who had an early COVID‐19 infection (i.e., an infection with wild‐type SARS‐CoV‐2 before September 2020), those that required hospitalization for their early infection were somewhat more likely to require hospitalization for their Omicron reinfection than those whose early COVID‐19 infection did not require hospitalization (4/61 [6.5%] vs. 1/116 [0.9%]; *p* = 0.09).

**TABLE 1:** Characteristics of cohort participants with symptomatic Omicron infection by early SARS‐CoV‐2 infection status.

	No early COVID‐19 (*N* = 84)	Early COVID‐19, 14–26 months before first Omicron infection (*N* = 88)	Early COVID‐19, > 26 months before first Omicron infection (*N* = 89)	Chi‐squared/Fisher *p*
Sex at birth, female (*N*, %)	48 (57)	48 (55)	55 (62)	0.61
Age in years at the start of Omicron era (median, IQR)	52 (36–66)	48 (34–58)	52 (42–63)	—
18–49 (*N*, %)	39 (46)	48 (55)	41 (46)	0.17
50–64 (*N*, %)	22 (26)	29 (33)	29 (33)	—
≥ 65 (*N*, %)	23 (27)	11 (12)	19 (21)	—
Underlying medical (non‐immunocompromising) comorbidity (*N*, %)	34 (40)	20 (23)	27 (30)	0.04a
Immunocompromised (*N*, %)	5 (6.0)	8 (9.1)	4 (4.5)	0.45
Number of vaccine doses before first Omicron infection (*N*, %)				
0, 1	3 (3.6)	7 (7.9)	1 (1.1)	< 0.001a,c
2, 3	61 (73)	80 (91)	65 (73)	—
≥ 4	20 (24)	1 (1.1)	23 (26)	—
Days since last vaccine dose^a^ (median, IQR)	157 (98–207)	151 (66–192)	254 (176–387)	—
0–119	27 (32)	35 (40)	15 (17)	< 0.001b,c
≥ 120	55 (65)	46 (52)	74 (83)	—
Neighbourhood income quintile (%)				
1 (lowest)	22 (26)	22 (25)	15 (17)	0.53
2	15 (18)	17 (19)	25 (28)	—
3	12 (14)	18 (20)	17 (19)	—
4	14 (17)	16 (18)	13 (15)	—
5 (highest)	21 (25)	15 (17)	19 (21)	—
Hand‐to‐face habits (*N*, %)				
None	11 (13)	18 (20)	21 (24)	0.45
1–6 times per day	58 (69)	53 (60)	51 (57)	—
≥ 6 times per day	15 (18)	17 (19)	17 (19)	—
Period of first Omicron infection^b^ (*N*, %)				< 0.001a,b,c
BA.1/2	39 (46)	79 (90)	0	
BA.4/5	36 (43)	9 (10)	57 (64)	—
BA.5/BQ1.1/XBB	9 (11)	0	32 (36)	—
Infections diagnosed by PCR vs. RAT (*N*, %)	36 (43)	48 (55)	34 (38)	0.08
Severity and fever at first omicron infection^a^				0.25
1: No fever, able to do regular ADL	23 (28)	35 (40)	19 (21)	—
2: Had fever, but was able to do regular ADL	13 (16)	17 (19)	14 (16)	—
3: No fever, not well enough to do regular ADL	15 (18)	13 (15)	19 (21)	—
4: Fever, and not well enough to do regular ADL	24 (29)	18 (20)	29 (33)	—
5: Bed bound or hospitalized^c^	8 (10)	5 (5.7)	8 (9.0)	—
Healthcare required for Omicron infection episode				
No	65 (78)	76 (87)	74 (83)	0.29
Yes	18 (22)	11 (13)	15 (17)	—
Time to full recovery^a^, days (median, IQR)	7.5 (6–13)	7 (4–10)	6 (5–10)	—
0–4 (*N*, %)	11 (13)	20 (23)	14 (16)	0.004a,c
5–9 (*N*, %)	31 (37)	39 (44)	36 (40)	—
10–14 (*N*, %)	12 (14)	5 (5.7)	14 (16)	—
15–19 (*N*, %)	1 (1.2)	8 (9.1)	1 (1.1)	—
≥ 20 (*N*, %)	11 (13)	7 (8.0)	4 (4.5)	—
Missing (*N*, %)	18 (21)	9 (10)	20 (22)	—
Two or more Omicron infections (*N*, %)	7 (8.3)	3 (3.4)	2 (2.3)	0.18

*Note:* Pairwise comparisons (a, b, c): (a) No early COVID‐19 versus early COVID at 14–26 months prior *p* < 0.05. (b) No early COVID‐19 and early COVID > 26 months prior *p* < 0.05. (c) Early COVID‐19 at 14–26 months and early COVID‐19 > 26 months prior *p* < 0.05.

Abbreviations: ADL = activities of daily living, IQR = interquartile range, PCR = polymerase chain reaction, RAT = rapid antigen test.

^a^ Distribution of missing values: *n* = 1 missing fever/ADL; *n* = 2 missing health care required; *n* = 47 missing duration of illness. Nine unvaccinated participants have no time from last vaccine dose (in multivariable analysis, these patients are grouped with those whose last dose was at > 120 days).

^b^ Omicron periods were defined based on Ontario's routine whole genome sequencing, as beginning the first week when more than 50% of sequenced viruses were of that variant and ending the last week when more than 50% of sequenced viruses were of another variant/sub‐variant [9]: BA.1/2: 12 December 2021 to 3 June 2022; BA.4/5: 4 June 2022 to 13 November 2022; BA.5/BQ1.1/XBB:14 November 2022 to 24 April 2023.

^c^ Overall, 4 of 61 reinfections in those hospitalized for COVID‐19 in 2020 compared to 1 of 106 infections managed as outpatients required hospitalization for their first Omicron infection (*p* = 0.09). None of 84 persons without an early infection required hospitalization for their first Omicron infection.

Having early COVID‐19 14–26 months prior was associated with a lesser impact on fever/ADL (OR = 0.50 [95% CI: 0.28, 0.87]; *p* = 0.02) for a subsequent Omicron infection compared to those without early COVID‐19. Those with early infection 14–26 months prior also had a trend towards a reduced number of healthcare interactions (OR = 0.48 [95% CI: 0.19, 1.2]) and shorter duration of illness (OR = 0.62 [95% CI: 0.33, 1.2]) compared to those without early COVID‐19; however, neither of these associations was statistically significant. Early COVID‐19 that occurred > 26 months prior to an Omicron infection did not appear to affect the severity of an Omicron infection (Table 2).

**TABLE 2:** Final adjusted^a^ multivariable model results for the association between early SARS‐CoV‐2 infection and severity of first Omicron infection.

Exposure	Impact on fever/ADL^b^		Healthcare required^c^		Duration of illness^d^	
	OR (95% CI)	*p*	OR (95% CI)	*p*	OR (95% CI)	*p*
History of symptomatic SARS‐CoV‐2 infection						
No early infection	Ref		Ref		Ref	—
Early infection 14–26 months before Omicron infection^e^	0.50 (0.28, 0.87)	0.02	0.48 (0.190, 1.24)	—	0.62 (0.33, 1.17)	
Early infection > 26 months before Omicron infection	1.23 (0.70, 2.15)	—	1.03 (0.43, 2.46)	—	0.65 (0.34, 1.25)	—
Immunocompromising conditions	6.51 (2.40,17.7)	< 0.001	11.8 (3.35, 41.5)	< 0.001	4.16 (1.50, 11.5)	0.01
Underlying chronic medical conditions	1.48 (0.88, 2.49)	—	3.24 (1.43, 7.34)	0.01	2.77 (1.50, 5.13)	0.001
Sex: Female	0.98 (0.61, 1.57)	—	1.01 (0.49, 2.10)	—	3.22 (1.83, 5.65)	< 0.001

*Note:* ‘—’ indicates *p* ≥ 0.10.

Abbreviations: ADL = activities of daily living, CI = confidence interval, IQR = interquartile range, OR = odds ratio, Ref = reference.

^a^ Adjusted models including the following covariates: age (continuous), sex (male or female), neighbourhood income quintile (from 1 = *highest income* to 5 = *lowest income*), underlying chronic medical conditions (any or none), immunocompromising conditions (any or none), time from most recent vaccine dose (0–119 or ≥ 120 days) and hand‐to‐face habits (0, 1–6 or ≥ 6 times per day). Impact on ADL and duration of illness were ordinal regression models with five‐level categorical outcomes; healthcare required was a binary logistic regression model.

^b^ Outcome was the impact of illness based on activities of daily living (ADL) including the presence of fever (no fever and able to do regular ADL, had fever but able to do regular ADL, no fever but not well enough to do regular ADL, had fever and not well enough to do regular ADL or bed bound or hospitalized) and modelled using proportional odds ordinal regression models.

^c^ Outcome was a requirement for healthcare (≥ 1 healthcare visit or none) and modelled using logistic regression.

^d^ Outcome was duration of illness defined as number of days from onset to full recovery (0–4, 5–9, 10–14, 15–19 or ≥ 20 days) and modelled using proportional odds ordinal regression models.

^e^ In those patients with early COVID‐19 (i.e., an infection before 30 September 2020), the median time from the early infection to their first Omicron infection was 793 days (26 months).

In sensitivity analyses, diagnosis method (PCR vs. RAT), Omicron infection period, and number of COVID‐19 vaccine doses and patient status in 2020 appeared unrelated to the main study outcomes. In the secondary analyses, each 90‐day reduction in time from early infection to Omicron reinfection was associated with lower odds of being in a higher severity category for the impact on fever and fever/ADL outcome: OR = 0.75 [95% CI 0.62, 0.91; *p* = 0.004] (Figure S5).

## 4 Discussion

In a highly vaccinated population, symptomatic SARS‐CoV‐2 infection early in the pandemic was associated with reduced severity of a first Omicron infection 14–26 months later. Protection from a prior infection appeared to last approximately 2 years, and declined over time. This suggests that continued efforts for SARS‐CoV‐2 prevention will likely be needed despite currently high levels (> 95%) of population seroprevalence.

Our results are consistent with previous studies using self‐reported symptoms that found lower risk and severity for reinfections after adjusting for vaccination status and SARS‐CoV‐2 variant [4, 11]. As reported elsewhere [11, 12], our data suggest that hospitalization for an early COVID‐19 episode may be associated with a higher odds of hospitalization for subsequent Omicron infection, indicating that these patients remain at increased risk for hospitalization for subsequent infections. In contrast to Hadley et al., substantially fewer of our participants hospitalized for early infection required hospitalization for subsequent Omicron infection (6% vs. 27%) [12].

The strengths of our study included validation of early COVID‐19 cases with SARS‐CoV‐2 testing, continuous follow‐up of patients, validated outcome measurements for ADL and objective measures of healthcare use and symptom duration. Limitations included the exploratory analysis and small sample size, which limited our power to conduct stratified analyses. Another limitation was that we evaluated only early symptomatic infections, which may not be generalizable to prior asymptomatic infections. Additionally, self‐reported outcomes to quantify illness severity used in our study have not been previously validated. Because time from early COVID‐19 to Omicron infection is correlated with the infecting variant, we cannot be sure that viral evolution, rather than time alone, contributed to reduced impact on the severity of later Omicron infections. Finally, our study was conducted in a single urban area with high COVID‐19 vaccination coverage and low pre‐Omicron infection rates, which may limit generalizability.

## 5 Conclusion

Having COVID‐19 early in the pandemic was associated with reduced severity of Omicron infections that occurred up to 26 months later. Protection provided by early infection, however, appeared to last only about 2 years—suggesting that continued efforts for SARS‐CoV‐2 prevention will likely be needed.

## Author Contributions

Conception and design: Ca.K., Z.Z., b.l.c., L.G., W.L.G., S.M., J.M.M. and A.M. Acquisition and analysis of data: Ca.K., A.S., Ch.K., L.F., Z.Z., A.‐C.G., b.l.c., L.G., T.M.,. S.M., C.M., M.H.K. and A.M. Interpretation of data: A.S., Ca.K., A.‐C.G., b.l.c., W.L.G., M.M., T.M., S.M., S.R.V., C.M., M.H.K., J.M.M. and A.M. Drafting of the manuscript: A.S., Z.Z., A.M. and Ch.K. Revision of manuscript: b.l.c., W.L.G., M.M., C.M., M.H.K. and J.M.M. All authors critically reviewed, revised and approved the submitted manuscript.

## Funding

This study was conducted as a collaboration between Sinai Health System and Pfizer. Sinai Health System was the study sponsor, with Pfizer (68616285), providing funding to Sinai Health System for protocol development, data generation and analysis and manuscript development.

## Ethics Statement

Informed consent was obtained from all study participants. The study was approved by the research ethics boards of participating hospitals (North York General #2024‐0233‐1025; Sunnybrook Health Sciences Centre #149‐1994; Michael Garron Hospital #084‐0209‐Lab‐01; University Health Network #14‐8339‐AE; Scarborough Health Network #MED‐02‐011; William Osler Health System #95‐0001; and Mount Sinai Hospital #21‐0069‐E).

## Conflicts of Interest

M.H.K., C.M., M.M., S.R.V. and J.M.M. are employees and shareholders of Pfizer Inc. A.M. declares grants and personal fees from Pfizer, grants from Sanofi and personal fees from AstraZeneca, GlaxoSmithKline, Moderna and Novavax, outside the submitted work. A.‐C.G. has received research funds from Providence Therapeutics Holdings Inc., outside the submitted work. The other authors declare no conflicts of interest.

## Supporting information




**Figure S1:** PCR confirmed cases of COVID‐19 Toronto, Canada, 2020–2021.
**Figure S2:** Hospitalization rates due to COVID‐19 in Toronto, Canada 2022–2023.
**Figure S3:** Flow chart of participant eligibility for severity analysis.
**Figure S4:** Distribution of duration of illness, first Omicron infection.
**Figure S5:** Severity of Omicron infection by time from early infection to first Omicron infection (time as continuous variable).
**Table S1:** Characteristics participants and infections for the 12 participants with more than one Omicron infection.

## Data Availability

The datasets analysed during the current study are available from the corresponding author on reasonable request.
