# Supplementary material for: Early COVID‐19 and Severity of Subsequent Omicron Infection in Ontario Canada
Source: Influenza Other Respir Viruses. 2026 Jan 12;20(1):e70197. doi: 10.1111/irv.70197 (PMC12795780; doi:10.1111/irv.70197)
Supplement: Supplementary file 1 — Figure S1: PCR confirmed cases of COVID‐19 Toronto, Canada, 2020–2021. Figure S2: Hospitalization rates due to COVID‐19 in Toronto, Canada 2022–2023. Figure S3: Flow chart of participant eligibility for severity analysis. Figure S4: Distribution of duration of illness, first Omicron infection. Figure S5: Severity of Omicron infection by time from early infection to first Omicron infection (time as continuous variable). Table S1: Characteristics participants and infections for the 12 participants with more than one Omicron infection. [file IRV-20-e70197-s001.docx]

**Supplementary Material**

**Kassee et al.**

**Early COVID-19 and Severity of Subsequent Omicron Infection in Ontario, Canada**

**Contents:**

**A. Setting and cohort development………………………………………………………page 1**

Supplementary Figure 1: PCR confirmed cases of COVID-19 Toronto, Canada, 2020-21

Supplementary Figure 2: Hospitalization rates due to COVID-19 in Toronto, Canada 2022-23

Supplementary Figure 3: Flow chart of participant eligibility for severity analysis

**B. Measures of severity of illness…………………………………………………………page 5**

Supplementary Figure 4: Distribution of duration of illness, first Omicron infection

**C. Supplementary Figure 5:** Severity of Omicron infection by time from early infection to first Omicron infection (time as continuous variable)………………………...……...….**page 7**

**D. Supplementary Table 1:** Characteristics participants and infections for the 12

participants with more than one Omicron infection……………………...……………....**page 8**

**A. Setting and Cohort Development**

*Setting*

This study was performed in Toronto, Canada. The first SARS-CoV-2 infection in Toronto was diagnosed in a traveler from China on January 23, 2020. Cases began increasing rapidly in March, and a lockdown was started on March 15, 2020. Materials and reagents for PCR testing for COVID-19 were limited until mid-June 2020, and testing was restricted to: patients requiring hospitalization, the first three illnesses in respiratory outbreaks in long term care homes, and out-patients who met at least one of three criteria: they were healthcare workers, they had travelled outside of Canada in the 14 days before onset of symptoms, or they were a close contact of a diagnosed case (Figure 1). By June of 2020, case rates had decreased and testing availability increased, and from June 15, 2020 to December 30, 2021, PCR testing was recommended and offered to all persons residing in Ontario.

Seroprevalence studies estimated that 1.3% of adults had been infected with SARS-CoV-2 by October 2020, 3.0% by February 2021 and 6.1% by the time of the onset of Omicron in December of 2021. (1)


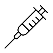

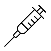

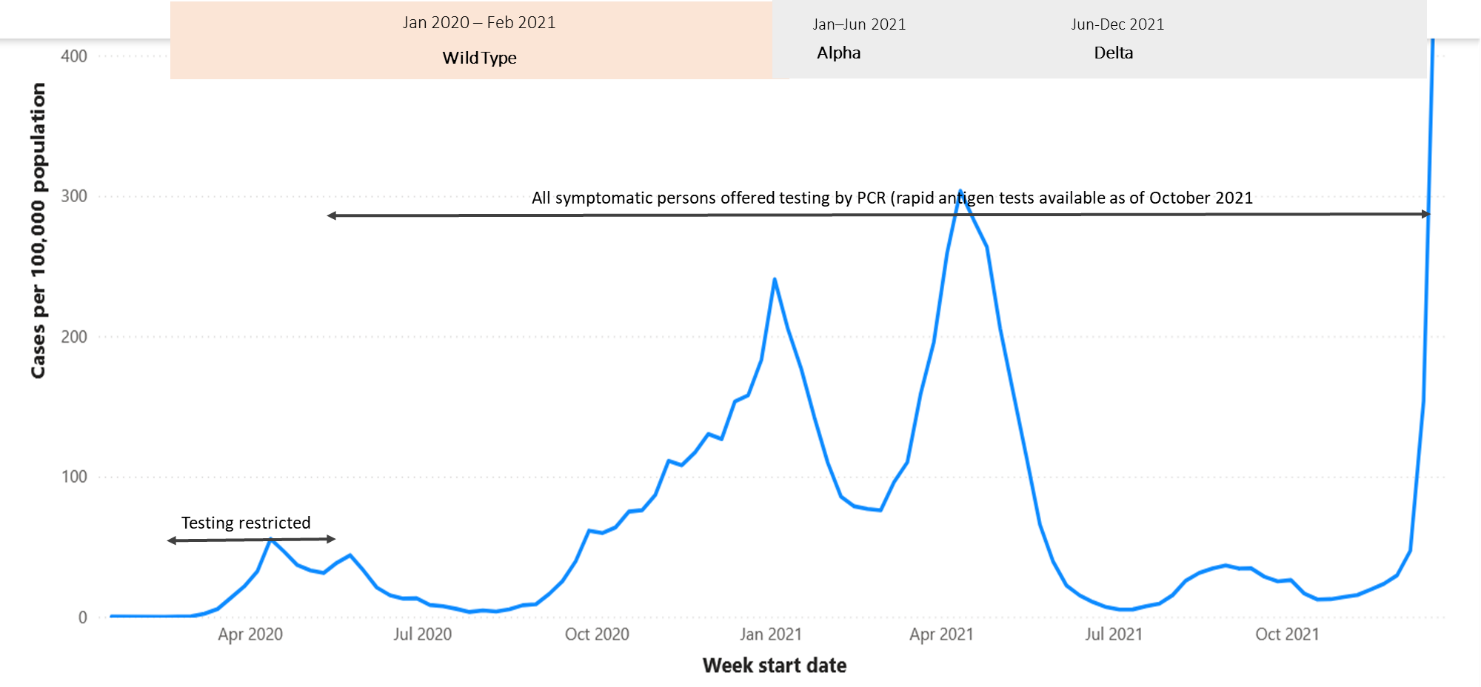


2021 Jan

Vaccine roll-out starts

2021 Nov

Booster #1 starts

Figure 1. PCR confirmed cases of COVID-19 over time in Toronto, Canada. As noted in text, testing was restricted from March to mid-June 2020, then available and recommended to all. Lockdown began on March 15, 2020, and public health restrictions at varying levels continued until mid-December 2021.(2) The Alpha variant was first detected in Ontario in January 2021 and became the dominant variant by April. In May 2021, routine sequencing of representative samples of SARS-CoV-2 variants was started in Ontario, and variant periods were subsequently defined based on this sequencing, as beginning the first week when more than 50% of sequenced viruses were of that variant, and ending the last week when more than 50% of sequenced viruses were of another variant/sub-variant (3).

The Omicron wave began in Ontario on December 12, 2021. By December 31, 2021, public health restrictions were re-introduced, and PCR testing was overwhelmed in the province such that routine PCR testing was no longer available for out-patients. Rapid antigen tests (RATS) were distributed free of charge at pharmacies and grocery stores as of February 9, 2022 (study participants had RAT kits distributed in November, 2021 and could also obtain specimens and submit them for PCR testing through the study).


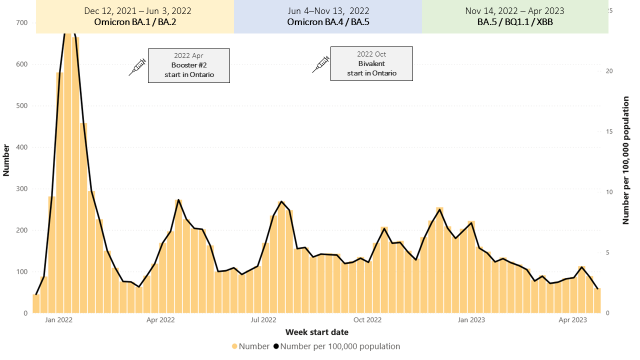


Figure 2: COVID-19 hospitalizations in Toronto during the Omicron variant period from December 12, 2021 to April 30, 2023. Variant periods are marked, as is the timing of first availability of updated vaccine doses.(2-4)

*Cohort Derivation*

The base cohort for this study included recruited participants from seven hospitals in Toronto, Canada. Eligible individuals were adults requiring hospitalization who tested positive for SARS-CoV-2 by PCR at or during admission between January 15 and September 30, 2020 and (N=1194) survived their admission, as well as a random selection of adult outpatients (N=1342) testing positive for SARS-COV-2 by PCR in emergency departments or COVID-19 assessment clinics between January 15 and June 15, 2020. These patients were recruited for studies of the course of infection, environmental contamination and measurement of acute and convalescent antibody titers (e.g. reference 5).

In the fall of 2021, members of these cohorts were re-approached about participation in a study of the impact of early COVID-19 infection on later COVID-19 (see also reference 6). Of the original cohorts, 740 inpatients and 745 outpatients had survived, could be contacted and agreed to participate in this second study. At the same time, we approached test negative controls, aiming to recruit 1 control for every 4 cases. For inpatients, we aimed to individually match 20% of cases, with matching by date of hospitalization and age (±10 years). For outpatients, we frequency matched 20% of cases by indication for testing (i.e. HCW, traveler, or contact of case), age group (quantiles of age), and date of testing (same month).

Overall, 1,123 individuals consented to participate: 895 cases: 414 inpatients, 481 outpatients, and 228 test negative controls: 119 inpatients, and 109 outpatients. Participants completed baseline questionnaires (on-line or by telephone) including demographic and health information, history of SARS-CoV-2 testing (with type of test) and results, COVID-19 vaccines received (including brand and date received) household composition, occupation, whether working from home or not, frequency of activities with potential exposure (e.g public transit use), and information regarding adherence to public health mitigation measures. They were then followed with biweekly surveys until January 31, 2023 (with a few continuing beyond this date at their request who are included in this analysis). Biweekly surveys collected data on episodes of respiratory illness, testing for SARS-CoV-2, new chronic medical conditions and medication, COVID-19 vaccine doses received, vaccination history (including dates and vaccine type). Illness reports were completed for all illness episodes, and included presence or absence of fever, impact on activities of daily living, healthcare utilization (number and type of visits) and illness duration.

A total of 800 participants of the 1123 (71%) had completed data for underlying illness, all bi-weekly follow-ups and number of vaccine doses received. Of these, 276 had had at least one Omicron infection. We excluded those participants who had had an infection after September 30, 2020 but before Omicron (ie. before December 12, 2021) (N=2), and those participants whose Omicron infections were asymptomatic (N=13). Asymptomatic Omicron infection were excluded from the analysis because, although we recorded their existence when identified, we did not systematically perform surveillance to identify them, such that the identified cases may not be representative of all asymptomatic illnesses. Thus, 261 participants were included in the final cohort for this analysis of severity of first Omicron infection.


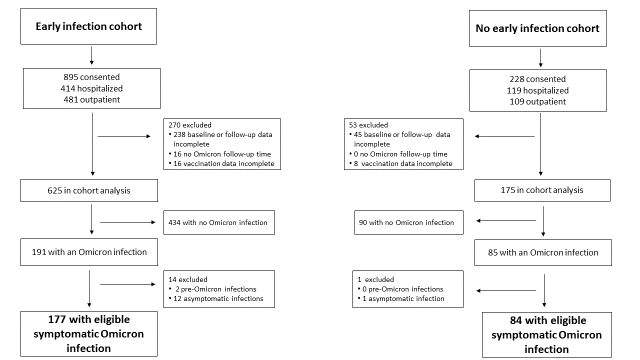


Figure 4: Flow chart of patients enrolled in the prospective cohort and eligible for the severity of illness analysis.

**References**

1. COVID_19 Immunity Task Forces. SARS-COV-12 seroprevalence in Canada. Available at: <https://www.covid19immunitytaskforce.ca/seroprevalence-in-canada/> [Accessed October 13, 2025]
2. Public Health Ontario. Enhanced Epidemiological Summary: COVID-19 Variants of Concern in Ontario: December 1, 2020 to May 9, 2021 Available at: <https://www.publichealthontario.ca/-/media/Documents/nCoV/Archives/VoC/covid-19-voc-report-2021-05-23.pdf?rev=fb548f6a776344569454676c4075ef70&sc_lang=en> [Accessed October 13, 2025]
3. Public Health Ontario. Archive of SARS-CoV-2 Whole Genome Sequencing in Ontario. Available at: <https://www.publichealthontario.ca/en/Data-and-Analysis/Infectious-Disease/COVID-19-Data-Surveillance/Archives/Whole-Genome-Sequencing>. [Accessed October 13, 2025].
4. Public Health Ontario. COVID-19 hospital admissions in Toronto Public Health, December 2021 to April 2023. Available at <https://www.publichealthontario.ca/en/Data-and-Analysis/Infectious-Disease/Respiratory-Virus-Tool>
5. Kotwa JD, Jamal AJ, Mbareche H, et al. Surface and Air Contamination With Severe Acute Respiratory Syndrome Coronavirus 2 From Hospitalized Coronavirus Disease 2019 Patients in Toronto, Canada, March-May 2020. J Infect Dis. 2022;225:768-776. doi: 10.1093/infdis/jiab578.
6. Shigayeva A, Kandel C, Farooqi L, et al. Early COVID-19 and protection from Omicron in a highly vaccinated population in Ontario, Canada: a matched prospective cohort study. BMC Infect Dis. 2025;25:194. doi: 10.1186/s12879-024-10331-1.

**B. Measures of severity of illness**

For this analysis, we were interested in the assessing the overall impact of illness, rather than the severity of individual symptoms. We also attempted to minimize the number of questions asked, given the demands this study made of participants during an already difficult time. Participants were asked the following questions:

1. How severe was this illness episode?

I tested positive but had no symptoms at all

I had symptoms, but was able to do regular activities on every day of the illness

I was not well enough to do regular activities on _______ days

I was not well enough to get out of bed for _______ days

I was admitted to the hospital for _______ days

2. What care did you require?

None

One or more visits with a health care provider, virtual or in-person

One or more visits to an emergency department

I was admitted to the hospital

3. Did you have a fever?

No and I did not feel feverish

I felt feverish but did not take my temperature

Yes

If yes,  What was the highest temperature measured _________

On how many days did you have a temperature of more than 38ºC? ___days

4. On what date did you feel completely recovered? Calendar drop down

Each of these outcomes was correlated with the others. The addition of fever to the ability perform ADL increased the correlation with care required and duration of illness over each variable alone and permitted a scale with an increased number of categories with face validity for severity of infection. Because 44 patients did not provide a number of days for the categories in question #1, we did not attempt to enhance the scale with duration of differing impact on activities of daily living (ADL).

The outcomes thus selected were:

A. An ordinal scale of ADL and fever combined:

1: No fever, able to do regular ADL

2: Had fever, but was able to do regular ADL on all days of illness

3: No fever, not well enough to do regular ADL

4: Fever, and not well enough to do regular ADL

5: Bed bound or hospitalized

B. Required at least one healthcare visit (Yes/No)

C. Duration of illness; categorized as per the figure below, because the distribution was not normal.

Figure 4: Distribution of duration of illness for the first Omicron infection in study participants, with categorization of outcomes **
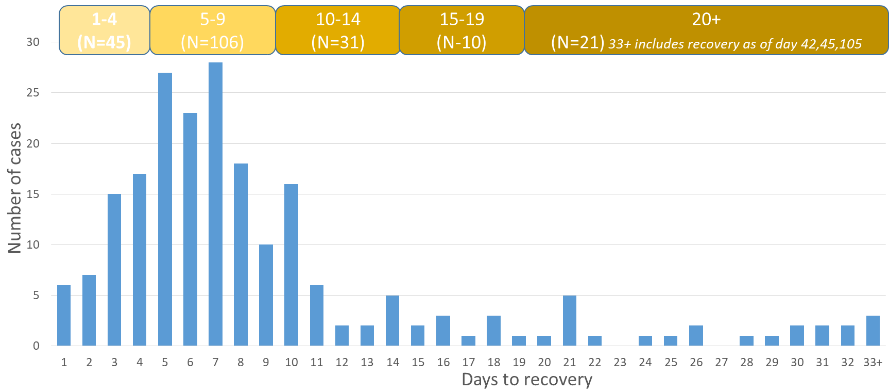
**

**C. Supplementary Figure 5:** Adjusted modelled probability of severity of Omicron infection (score based on presence/absence of fever and impact on activities of daily living) by time (number of days) from early infection to first Omicron infection. Blue shading indicates 95% confidence limits.

A. Probability of being bed bound or hospitalized;

B. Probability of having a fever and not being well enough to do regular ADL or more severe (ie. of being bed-bound or hospitalized).

C. Probability of no fever but not being able to do regular ADL or more severe;

D: probability of having symptoms and fever, but able to perform regular ADL or more severe. Each 90-day reduction in time from early infection to Omicron reinfection was associated with lower odds of being in a higher severity category, for the impact on fever and ADL outcome: OR=0.77 [95% CI 0.63, 0.94; *P*=.009]

A

B

C

D

**D. Supplementary Table 1:** Characteristics of participants and infections for the 12 participants with more than one Omicron infection

| Age  (yrs) | Sex | At least one co-morbidity | Immuno-compromised | Early COVID-19 | First Omicron  Infection Period† | Number of COVID-19  Doses Before 1^st^ Omicron infection | 2^nd^ Omicron  Infection  Period | Total Number of COVID-19  Vaccine Doses | ADL-Fever  first vs second infection‡ | Health care required first/ second infection§ | Number of days  until full recovery  first/second infection¶ |
| --- | --- | --- | --- | --- | --- | --- | --- | --- | --- | --- | --- |
| 66 | Female | No | No | Yes | BA.1/2 | 3 | BA.4/5 | 3 | 1/1 | 0/0 | 7/7 |
| 63 | Male | Yes | No | No | BA.1/2 | 2 | BA.5/BQ.1.1/XBB | 2 | 1/1 | 0/0 | 2/8 |
| 32 | Female | No | No | Yes | BA.4/5 | 3 | BA.5/BQ.1.1/XBB | 4 | 1/2 | 0/0 | NA/3 |
| 60 | Female | No | No | No | BA.1/2 | 3 | BA.5/BQ.1.1/XBB | 3 | 1/4 | 0/0 | 2/9 |
| 37 | Female | No | No | Yes | BA.1/2 | 2 | BA.5/BQ.1.1/XBB | 2 | 1/4 | 0/0 | 8/NA |
| 51 | Male | Yes | No | Yes | BA.1/2 | 3 | BA.5/BQ.1.1/XBB | 3 | 2/1 | 0/0 | 4/7 |
| 43 | Female | Yes | No | No | BA.1/2 | 3 | BA.5/BQ.1.1/XBB | 4 | 2/5 | 0/1 | 30/NA |
| 30 | Female | Yes | No | No | BA.4/5 | 3 | BA.5/BQ.1.1/XBB | 3 | 3/2 | 0/0 | 6/5 |
| 63 | Female | Yes | No | Yes | BA.4/5 | 4 | BA.5/BQ.1.1/XBB | 4 | 3/3 | 0/0 | 2/8 |
| 38 | Male | No | Yes | No | BA.4/5 | 3 | BA.5/BQ.1.1/XBB | 4 | 3/3 | 0/0 | 13/8 |
| 66 | Male | Yes | No | No | BA.1/2 | 3 | BA.5/BQ.1.1/XBB | 5 | 4/2 | 1/0 | 3/5 |
| 53 | Female | Yes | No | No | BA.1/2 | 3 | BA.4/5 | 4 | 5/4 | 1/1 | NA/NA |

Abbreviation: ADL=activities of daily living; NA=not available; yrs=years

† Omicron periods were defined based on Ontario’s routine whole genome sequencing, as beginning the first week when more than 50% of sequenced viruses were of that variant, and ending the last week when more than 50% of sequenced viruses were of another variant/sub-variant.[13]: BA.1/2**:** 12-Dec-2021 to 03-June-2022; BA.4/5: 04-June-2022 to 13-Nov-2022; BA.5/BQ1.1/XBB:14-Nov-2022 to 24-April-2023

‡Scores for this outcome are: 1=no fever, able to perform all ADL, 2=fever, but able to perform all ADL, 3=no fever, not able to perform all ADL; 4=fever and not able to perform all ADL, 5=bedbound or hospitalized; Wilcoxon rank sum test comparing the two infections P=0.52

§Scores for this outcome are: 0=no healthcare required; 1=at least one visit with a healthcare provider required. Wilcoxon rank sum test comparing the two infections P=1.0

¶Wilcoxon rank sum test comparing the two infections P=1.0
